# Sex and age disparities in the influence of tobacco smoking on depression: Evidence from the Taiwan Biobank

**DOI:** 10.18332/tid/174643

**Published:** 2023-11-24

**Authors:** Shaw-Ji Chen, Meng-Ying Lu, Jerry Cheng-Yen Lai, Ming-Jong Bair, Hsiao-Yang Cheng

**Affiliations:** 1 Department of Psychiatry, MacKay Memorial Hospital, Taitung, Taiwan; 2 Department of Medicine, Mackay Medical College, New Taipei City, Taiwan; 3 National Taitung University, Taitung, Taiwan; 4 Division of Cardiology, Department of Internal Medicine, MacKay Memorial Hospital, Taitung, Taiwan; 5 Department of Medical Research, MacKay Memorial Hospital, Taitung, Taiwan; 6 Division of Gastroenterology, Department of Internal Medicine, MacKay Memorial Hospital, Taitung, Taiwan

**Keywords:** Taiwan Biobank, depression, tobacco, sex, age

## Abstract

**INTRODUCTION:**

The global tobacco epidemic poses a notable challenge to global health due to its association with various tobacco-related diseases. Although tobacco smoking is associated with depression, the exact mechanism by which tobacco smoking increases the risk of depression is unclear. This study explored the potential effects of tobacco smoking on depression.

**METHODS:**

We used data in the analysis from the Taiwan Biobank of 27916 individuals recruited from 2015 to 2020. To investigate the associations between tobacco use and depression, the results of the depression-measuring subscale of the Patient Health Questionnaire-4 as well as data on participants’ tobacco consumption and other relevant covariates, were analyzed.

**RESULTS:**

Participants who smoked were more likely to report depression than those who did not smoke (AOR=1.50; 95% CI: 1.21–1.86). Furthermore, depression was significantly higher in women who smoked than in their male counterparts (females: AOR=1.68; 95% CI: 1.27–2.23, and males: AOR=1.32; 95% CI: 0.96–1.80). Women aged <55 years and who smoked were more likely to report depression, whereas this trend was not observed in those aged ≥55 years (<55 years: AOR=1.75; 95% CI: 1.23–2.48), and ≥55 years: AOR=1.58; 95% CI: 0.97–2.56).

**CONCLUSIONS:**

Tobacco smoking is a significant factor associated with depression, particularly in younger women. The increasing prevalence of tobacco use for years among younger women in Taiwan might contribute to shifts in the associations between depression and tobacco use in women.

## INTRODUCTION

The global tobacco epidemic poses a notable challenge to global health, as evidenced by its association with various tobacco-related diseases^1^. Tobacco smoking is associated with an increased risk of mental illness^2^. Depression is one of the most prevalent mental ailments worldwide, and it has imposed an increasing economic burden in recent years^3^. Between 2010 and 2018, the population of adults in the United States with major depressive disorder (MDD) increased by 12.9%. Notably, the economic burden associated with the increased prevalence of MDD in the adult population increased by 37.9%^4^. The impact of depression encompasses not only its symptomatic aspects but also the impairment of occupational or educational functioning caused by the disorder and the care burden of family and friends. Although the relationship between depression and tobacco use has been demonstrated, the precise influence of tobacco use on depression is not well understood^5^. Tobacco use can increase the risk of depression or lead to the further use of tobacco as self-medication to combat depression. The shift in tobacco consumption trends in recent years is noteworthy, with smoking becoming increasingly prevalent among women, especially younger women^6^. Consequently, understanding the factors contributing to both tobacco smoking and depression is imperative. In addition to tobacco smoking, alcohol, coffee, and tea are commonly consumed, and the possible connections with substance use disorder have been noted^7-10^.

Numerous studies have investigated various risk factors for depression, and their results revealed that the risk of depression is influenced by an interplay of complex factors such as age, sex, body mass index (BMI), sleep, physical exercise, education level, residential area, and family history. Aging has been demonstrated to cause a modest increase in depressive symptoms in both men and women^11^. In terms of sex, women have been reported to have a higher prevalence of depression than men; however, the results have been inconsistent^12^. A higher BMI is associated with a higher risk of depression^13^. The sleep pattern has been a suspected contributor to depression^14^, with emerging interest in aspects beyond the total sleep time and sleep quality. The concept of social jet lag (SJL), which refers to the differences in sleep patterns between weekdays and weekends, was found to be related to psychiatric disorders, including depression^15^. Regular physical exercise has attracted attention for its potential preventive or therapeutic effects against depression^16^. Additionally, a higher education level has been demonstrated to exert a protective effect against depression, but this relationship remains underexplored^17^. Furthermore, a family history of depression has been recognized as a risk factor for depression onset and severity^18^.

This study explored the potential impact of tobacco smoking on depression. Our research was conducted using the extensive data available in the Taiwan Biobank (TWB), and the data aided in the identification of influence between tobacco smoking and depression.

## METHODS

We conducted a population-based, pooled, cross-sectional study using information on 33741 participants joining the TWB database during the years 2015 through 2020. To protect the participants’ confidentiality, de-identified data were received and used without alteration. The TWB has been established as a government-supported initiative aimed at obtaining lifestyle and genetic data from the Taiwanese population^19,20^. The recruitment process for the TWB involves enlisting community-based volunteers aged 30–70 years who have no history of cancer. After providing written informed consent, individuals participating in the TWB provided information pertaining to their daily lives, provided blood samples, and underwent a physical examination. In addition to blood sample collection and physical examination, each participant was required to complete a questionnaire through a face-to-face interview with one of the TWB researchers. The questions were related to personal information and lifestyle factors.

### Depression measurement

In the TWB, depression tendencies were assessed using the Patient Health Questionnaire-4 (PHQ-4)^19^. PHQ-4 is a short self-report 4-item questionnaire that contains two subscales including a 2-item anxiety scale (GAD-2) with the first two questions and a 2-item depression scale (PHQ-2) with the last two questions. A total score of ≥3 for the last two questions of the questionnaire indicated the presence of depression. This established criterion was employed in our study for evaluating depression^20^.

### Tobacco smoking

The study also conducted a survey of tobacco smoking which was divided into non-smoking and smoking groups. Smoking was defined as regularly engaging in smoking for a minimum duration of 6 months and not quitting smoking prior to completing the PHQ-4 questionnaire.

### Covariates in this study

During the interview for the TWB, a range of demographic data were collected including sex (male vs female); age (≥55 vs <55 years); BMI (≥25 vs <25 kg/m^2^); regular exercise (yes vs no); education level (college or graduate school vs high school, elementary school, or none); alcohol, coffee, and tea consumption (yes vs no); sleep patterns such as total sleep duration on weekdays compared to weekend, sleep differences between weekdays and weekend, and sleep quality; and family history of mental illness. For sleep quality data, the original categories of very good, good, average, bad, and very bad were dichotomized into: Good^+^ (very good, good, and average) and Bad^+^ (bad and very bad). During the interview for the TWB, the BMI (kg/m^2^) was measured^2^. These data were considered covariates for subsequent analyses according to the methods described in previous studies^7,12-18,21^.

We also recorded the occurrence of menopause among women who were part of the TWB and were aged >40 years.

### Statistical analysis

Characteristics of participants, with and without tobacco smoking behaviors, are expressed using means and standard deviation for continuous data, frequencies and percentages for categorical data, and odds ratios (ORs) with 95% confidence intervals. Continuous variables were compared using Student’s t-test, and categorical data were compared using the χ^2^ test or Fisher’s exact test. Participant characteristics with a p<0.05 were included in the logistic regression model, which was used to estimate covariate-adjusted odds ratios (AORs) for the possible association with depression. We analyzed all data using the Windows version 9.4 of SAS (Statistical Analysis System) (SAS Institute Inc.; Cary, NC). A p<0.05 was considered statistically significant.

## RESULTS

### Basic characteristics of TWB participants

**Figure 1:**
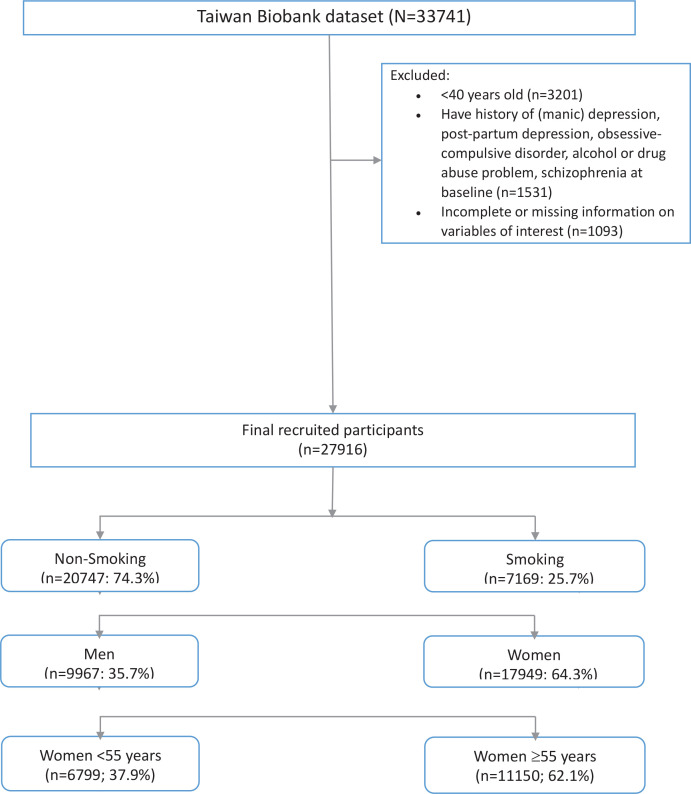
Population selection flowchart

After excluding 3201 participants aged <40 years from our study and those with incomplete information on independent variables of interest (n=1093), a total of 27916 respondents, comprising 17949 women (64.3%) and 9967 men (35.7%) were included in the data analysis. All participants were classified into two groups based on their smoking status: non-smoking (n=20747; 74.3%) and smoking (n=7169; 25.7%), comprising 4001 (19.3%) and 5966 (83.2) men, respectively. The participant selection process is shown in Figure 1.

Non-smokers had a mean age of 57.3 years (SD=8.8) and smokers 56.9 years (SD=8.9). Non-smokers aged <55 years were 7572 (36.5%) and smokers were 2818 (39.3%). Those having depression based on PHQ-4 scores were 497 (2.4%) and 200 (2.8%) in the non-smoking and smoking groups, respectively. All their details, including PHQ-4, tobacco use, sex, age, BMI, regular exercise, alcohol, coffee and tea consumption, education level, rural residence, family history, and sleep profiles, are presented in Table 1.

**Table 1:** Baseline characteristics, stratified by smoking behaviors, of participants aged 40–70 years, 2015–2020 (N=27916)

*Characteristics*	*Non-Smoking n (%)*	*Smoking n (%)*	*p*
**Total**	20747 (74.3)	7169 (25.7)	
**Male**	4001 (19.3)	5966 (83.2)	<0.001***
**Age** (years), mean (SD)	57.26 (8.79)	56.86 (8.91)	<0.001***
**Age** (years)			
<55	7572 (36.5)	2818 (39.3)	<0.001***
≥55	13175 (63.5)	4351 (60.7)	
**Residence**			
Rural	1146 (5.5)	505 (7.0)	<0.001***
Non-rural	19601 (94.5)	6664 (93.0)	
**Education level**			
College or graduate school	11107 (53.5)	3513 (49.0)	<0.001***
High school, elementary school or none	9640 (46.5)	3656 (51.0)	
**BMI (kg/m^2^)**, mean (SD)	24.03 (3.57)	25.27 (3.51)	<0.001***
**BMI** (kg/m^2^)			
<25	13625 (65.7)	3555 (49.6)	<0.001***
≥25	7122 (34.3)	3614 (50.4)	
**Behavioral factors**			
Regular exercise	10692 (51.5)	3441 (48.0)	<0.001***
Alcohol drinking	858 (4.1)	2222 (31.0)	<0.001***
Coffee drinking	8563 (41.3)	3400 (47.4)	<0.001***
Tea drinking	3976 (19.2)	2451 (34.2)	<0.001***
**Total sleep hours**			
Weekdays, mean (SD)	6.58 (1.10)	6.66 (1.12)	<0.001***
Weekend, mean (SD)	6.92 (1.31)	7.03 (1.36)	<0.001***
**Difference in sleep hours between weekdays and weekend**			
Difference in sleep hours, mean (SD)	0.37 (0.83)	0.42 (0.94)	<0.001***
Difference in sleep hours ≥2 hours	1972 (9.5)	825 (11.5)	<0.001***
**Sleep quality**			
Very bad + Bad (Bad^+^)	4223 (20.4)	1338 (18.7)	0.002**
Average + Good + Very good (Good^+^)	16524 (79.6)	5831 (81.3)	
Very good	2866 (13.8)	1198 (16.7)	<0.001***
Good	4327 (20.9)	1569 (21.9)	
Average	9331 (45.0)	3064 (42.7)	
Bad	3686 (17.8)	1180 (16.5)	
Very bad	537 (2.6)	158 (2.2)	
**Depression**			
PHQ-4 score, mean (SD)	0.72 (1.44)	0.69 (1.48)	0.115
Depression in PHQ-4	497 (2.4)	200 (2.8)	0.072
Family history of depression	596 (2.9)	134 (1.9)	<0.001***

BMI: body mass index. PHQ-4: patient health questionnaire-4.

* p<0.05

** p<0.01

*** p<0.001 (p-values, χ^2^ test or two-sample t test).

### Associations of smoking behaviors and depression

Association between tobacco use and depression variables that exhibited significance (p<0.05) in the univariate analysis were selected as covariates in the subsequent logistic regression analysis. The results revealed that participants who smoked exhibited a higher likelihood of depression than the reference group of non-smoking participants (AOR=1.50; 95% CI: 1.21–1.86). Other covariates that were associated with a higher likelihood of depression included female sex (AOR=1.64; 95% CI: 1.32–2.04) and a family history of depression (AOR=1.56; 95% CI: 1.06–2.29). Conversely, participants who were aged ≥55 years (AOR=0.67; 95% CI: 0.56–0.78), regularly exercised (AOR=0.61; 95% CI: 0.52–0.72), and had good sleep quality (AOR=0.29; 95% CI: 0.25–0.33) exhibited a lower likelihood of depression (Table 2).

**Table 2:** Associations of smoking behaviors and depression, in participants aged 40–70 years, 2015–2020 (N=27916)

*Variables*	*AOR (95% CI)*	*p*
Smoking (vs non-smoking)	1.50 (1.21–1.86)	<0.001***
Female (vs male)	1.64 (1.32–2.04)	<0.001***
Age (vs <55 years)	0.67 (0.56–0.78)	<0.001***
Rural residence (vs non-rural)	1.10 (0.79–1.53)	0.577
College or graduate school (vs high school, elementary school or none)	1.03 (0.88–1.21)	0.703
BMI (vs <25 kg/m^2^)	1.02 (0.87–1.20)	0.790
Regular exercise (vs No)	0.61 (0.52–0.72)	<0.001***
Alcohol drinking (vs No)	1.24 (0.96–1.60)	0.103
Coffee drinking (vs No)	0.99 (0.85–1.16)	0.924
Tea drinking (vs No)	0.89 (0.74–1.08)	0.229
Difference in sleep hours between weekdays and weekend (vs <2 hours)	1.07 (0.85–1.34)	0.562
Sleep quality (vs Bad^+^)	0.29 (0.25–0.33)	<0.001***
Family history of depression (vs No)	1.56 (1.06–2.29)	0.024*

AOR: adjusted odds ratio; covariates other than tobacco smoking were adjusted for in the statistical analysis. BMI: body mass index.

* p<0.05

** p<0.01

*** p<0.001.

### Sex-stratified associations between tobacco use and depression

In subsequent analyses, male and female participants were separately analyzed due to the increased odds of depression among female participants. Among female participants, those who smoked exhibited significantly higher odds of depression (AOR=1.68; 95% CI: 1.27–2.23) than male participants who smoked. Across both sexes, being aged ≥55 years (females: AOR=0.56; 95% CI: 0.41–0.76, and males: AOR=0.62; 95% CI: 0.48–0.81), having regular exercise (females: AOR=0.53; 95% CI: 0.39–0.74, and males: AOR=0.64; 95% CI: 0.53–0.78) and Good^+^ sleep quality (females: AOR=0.29; 95% CI: 0.22–0.39, and males: AOR=0.28; 95% CI: 0.24–0.34) were associated with reduced odds of depression (Table 3).

**Table 3:** Associations of smoking behaviors and depression, stratified by sex, in participants aged 40–70 years, 2015–2020 (N=27916)

*Variables*	*Men*		*Women*	
	*AOR (95% CI)*	*p*	*AOR (95% CI)*	*p*
Smoking (vs non-smoking)	1.32 (0.96–1.80)	0.089	1.68 (1.27–2.23)	<0.001***
Age (vs <55 years)	0.56 (0.41–0.76)	<0.001***	0.62 (0.48–0.81)	<0.001***
Post-menopausal (vs pre-menopausal)			1.26 (0.96–1.65)	0.092
Rural residence (vs non-rural)	0.97 (0.56–1.66)	0.910	1.16 (0.76–1.76)	0.493
College or graduate school (vs high school, elementary school or none)	0.86 (0.64–1.16)	0.320	1.14 (0.95–1.38)	0.166
BMI (vs <25 kg/m^2^)	1.02 (0.76–1.36)	0.898	1.03 (0.85–1.24)	0.794
Regular exercise (vs No)	0.53 (0.39–0.74)	<0.001***	0.64 (0.53–0.78)	<0.001***
Alcohol drinking (vs No)	1.10 (0.80–1.52)	0.549	1.49 (0.99–2.25)	0.057
Coffee drinking (vs No)	1.03 (0.77–1.38)	0.837	0.98 (0.82–1.18)	0.858
Tea drinking (vs No)	0.94 (0.69–1.29)	0.719	0.87 (0.68–1.10)	0.246
Difference in sleep hours between weekdays and weekend (vs <2 hours)	1.10 (0.72–1.67)	0.672	1.07 (0.81–1.40)	0.642
Sleep quality (vs Bad^+^)	0.29 (0.22–0.39)	<0.001***	0.28 (0.24–0.34)	<0.001***
Family history of depression (vs No)	1.77 (0.71–4.42)	0.224	1.52 (0.99–2.32)	0.054

AOR: adjusted odds ratio; covariates other than tobacco smoking were adjusted for in the statistical analysis. BMI: body mass index.

* p<0.05

** p<0.01

*** p<0.001.

### Associations between tobacco use and depression across age groups in females

To further refine the analysis, female participants were divided into two subgroups based on age: those aged ≥55 years and those <55 years. This division was based on the fact that women aged <55 years exhibited significantly higher odds of depression (AOR=1.75; 95% CI: 1.23–2.48). Although female participants aged ≥55 years who smoked did not exhibit significant odds of depression, those aged ≥55 years who consumed alcohol or had a family history of depression, had higher odds of depression (alcohol drinking: AOR=2.10; 95% CI: 1.23–3.59, and family history of depression: AOR=1.99; 95% CI: 1.16–3.41). Furthermore, regardless of age group, engaging in regular exercise (aged <55 years: AOR=0.71; 95% CI: 0.53–0.96, and aged ≥55 years: AOR=0.60; 95% CI: 0.46–0.77) and possessing Good+ sleep quality (aged <55 years: AOR=0.29; 95% CI: 0.22–0.39, and aged ≥55 years: AOR=0.27; 95% CI: 0.21–0.35) were associated with reduced odds of depression (Table 4).

**Table 4:** Associations of smoking behaviors and depression among the female population, stratified by age, in participants aged 40–70 years, 2015–2020 (N=17949)

*Variables*	*Aged <55 years*		*Aged ≥55 years*	
	*AOR (95% CI)*	*p*	*AOR (95% CI)*	*p*
Smoking (vs non-smoking)	1.75 (1.23–2.48)	0.002**	1.58 (0.97–2.56)	0.063
Post-menopausal (vs pre-menopausal)	1.22 (0.92–1.61)	0.169	3.61 (0.50–26.02)	0.202
Rural residence (vs non-rural)	0.94 (0.57–1.57)	0.819	1.70 (0.79–3.65)	0.174
College or graduate school (vs high school, elementary school or none)	1.18 (0.91–1.54)	0.217	1.11 (0.84–1.45)	0.471
BMI (vs <25 kg/m^2^)	1.07 (0.82–1.40)	0.624	0.98 (0.74–1.29)	0.876
Regular exercise (vs No)	0.71 (0.53–0.96)	0.028*	0.60 (0.46–0.77)	<0.001***
Alcohol drinking (vs No)	1.02 (0.54–1.94)	0.955	2.10 (1.23–3.59)	0.006**
Coffee drinking (vs No)	0.86 (0.66–1.11)	0.241	1.14 (0.88–1.49)	0.323
Tea drinking (vs No)	0.97 (0.70–1.33)	0.845	0.74 (0.51–1.08)	0.117
Difference in sleep hours between weekdays and weekend (vs <2 hours)	1.15 (0.84–1.56)	0.379	0.83 (0.46–1.51)	0.539
Sleep quality (vs Bad^+^)	0.29 (0.23–0.38)	<0.001***	0.27 (0.21–0.35)	<0.001***
Family history of depression (vs No)	1.08 (0.54–2.14)	0.833	1.99 (1.16–3.41)	0.013*

AOR: adjusted odds ratio; covariates other than tobacco smoking were adjusted for in the statistical analysis. BMI: body mass index.

* p<0.05

** p<0.01

*** p<0.001.

## DISCUSSION

We investigated the impact of tobacco smoking on depression by analyzing data retrieved from the TWB. Several noteworthy findings were obtained. First, we revealed that participants who smoked tobacco exhibited higher odds of depression than those who did not. Furthermore, we conducted sex-specific analyses and found that female participants who smoked tobacco had significantly higher odds of reporting depression than those who did not. Finally, a subgroup analysis of age-stratified groups of female participants revealed that younger women with a tobacco smoking habit exhibited higher odds of depression than older women.

The correlation between tobacco use and depression was established in a previous study^22^. The present study further confirmed this relationship. Additionally, compared with men, women exhibited higher odds of reporting depression. Therefore, in subsequent analyses, we evaluated the differences in the outcomes between men and women. We found that men who smoked tobacco did not exhibit significantly higher odds of reporting depression than women smokers. This finding may be attributed to the fact that the prevalence rate of tobacco smoking among the male participants was >60%, whereas that among female participants was <7% in our study. Notably, despite this disparity, our findings indicate a substantial association between tobacco smoking and depression among female participants. This finding is noteworthy considering the increasing prevalence of tobacco smoking among young women in recent years compared with older women^9^. A study demonstrated that tobacco smoking increased the risk of depression in women, with this trend more commonly observed in pre-menopausal than in post-menopausal women^23^. Furthermore, the younger women in our study, but not the older women, exhibited higher odds of reporting depression, although menopause was not identified as a significant factor for depression. This may be because of the complex interplay between the post-menopausal and aging effects on depression.

Covariates other than tobacco smoking were adjusted for in the statistical analysis. Among these covariates, regular exercise and good sleep quality emerged as protective factors in our study, which is in agreement with the results of previous studies. Regular physical exercise was identified as a protective factor and was found to reduce the prevalence of depression and potentially mitigate its symptoms^24^. In our study, the protective effect of regular exercise on depression was also significant across sex-stratified groups. Furthermore, in previous studies, poor sleep quality was associated with an increase in the OR for depression and other mental disorders. Moreover, in the present study, good sleep quality served as the protective factor against depression. A family history of depression was identified as a risk factor for depression in previous studies^18^, and the same result was observed for all the participants of the present study. Notably, in the present study, a family history of depression significantly increased the risk of depression in older women, but not in younger women. Additionally, older women who consumed alcohol exhibited significantly higher risk of depression. The same connection between alcohol use and family history of depression was noted in women in previous studies^25^. However, this phenomenon was only apparent in older women in the present study because the effect of tobacco smoking on depression was more pronounced in younger women than in older women^25^.

A potential link between tobacco smoking or alcohol use and depression has been suspected^26^. A prevailing hypothesis is the stress-coping model, which posits that individuals who experience higher levels of stress are more prone to resort to the use of tobacco, alcohol or other substances as a means of emotional regulation^27^. Older women were reported to consume alcohol to regulate emotions, especially after menopause^28^. In our study, we observed varying impacts of tobacco and alcohol use on the risk of depression among women across different age groups. This phenomenon could be indicative of the increased prevalence of tobacco smoking among young women in recent years.

### Limitations

This study has some limitations that must be acknowledged. First, we used PHQ-4 for evaluating depression among the participants of our study. However, PHQ-4 is a measure of merely the presence of depression symptoms and cannot be used to arrive at clinical diagnoses. Therefore, clinical confirmation through in-depth assessment is necessary to establish a definitive diagnosis of depression. Second, we only recruited participants aged >40 years from the TWB. Moreover, considering the recent shift in tobacco smoking trends, particularly among young adults, the study may not fully reflect the conditions and trends among young adults in Taiwan^29^. Third, this is a cross-sectional study which is not able to make causal inference between tobacco and depression. Forth, the exclusive use of TWB participants in the present study limits generalizability to other countries.

## CONCLUSIONS

This study provided valuable insights into the relationship between tobacco smoking and the odds of reported depression. A notable association was observed between habitual tobacco use and a high risk of depression. However, women, particularly younger women, had high odds of reporting depression. The prevalence rates of tobacco smoking tend to be considerably higher in men than in women. However, these differences between women and men have been decreasing over time^6^. Moreover, the prevalence of tobacco smoking has been increasing among younger women than among older women in recent years^30^. Given the increased risk of depression associated with tobacco smoking, especially among younger women, efforts should be taken to promote the cessation of tobacco smoking.

## Data Availability

The data supporting the findings of this study are available upon reasonable request from the TWB. The TWB restricts public access to its data.
